# Association between oral health and colorectal adenoma in a screening population

**DOI:** 10.1097/MD.0000000000012244

**Published:** 2018-09-14

**Authors:** Donghyoun Lee, Kyung Uk Jung, Hyung Ook Kim, Hungdai Kim, Ho-Kyung Chun

**Affiliations:** a Department of Surgery, Jeju National University Hospital, Jeju National University School of Medicine, Jeju Self-governing Province; b Department of Surgery, Kangbuk Samsung Hospital, Sungkyunkwan University School of Medicine, Seoul, South Korea.

**Keywords:** colonoscopy, colorectal adenoma, oral health, periodontitis

## Abstract

Although periodontal disease and gastrointestinal tract health are closely associated, few studies have investigated whether periodontitis is a risk factor for colorectal adenoma. The aim of this study was to investigate whether there is an association between periodontitis and the risk of colorectal adenoma in asymptomatic healthy people.

From January 2013 to October 2015, we retrospectively enrolled 42,871 patients who underwent health screening at Kangbuk Samsung Hospital in South Korea. Demographic and clinical data were collected before colonoscopy. We calculated the odds ratio (OR) for adenoma in these patients.

The median age was 39.3 ± 8.7 years and 70.4% of the patients were men; 32.5% had a body mass index (BMI) 25.0 kg/m^2^. The frequency of adenoma was 12% (n = 5136). A higher risk of adenoma was associated with the following factors: BMI 25.0 kg/m^2^ (OR 1.51, 95% confidence interval [CI]: 1.42–1.61), current smoker (OR 1.51, 95% CI: 1.42–1.61), former smoker (OR 1.28, 95% CI: 1.19–1.37), periodontitis (OR 1.95, 95% CI: 1.82–2.0), moderate alcohol intake (OR 1.8, 95% CI: 1.69–1.93), and heavy alcohol intake (OR 2.67, 95% CI: 2.24–3.18).

Being male or a former or current smoker, alcohol intake above the moderate level, and periodontitis increase the risk of colorectal adenoma. These findings suggest that controlling oral disease is important to the prevention and management of colorectal adenoma. The findings of this study could be applied to risk stratification and colorectal cancer prevention programs, including screening guidelines.

## 1 Introduction

Colorectal cancer (CRC) is the 3rd most common cancer and 4th leading cause of cancer-related death in worldwide.^[1]^ Because >90% of CRC cases involve adenocarcinoma that develops from an adenoma originating from colorectal mucosa, screening for and early identification, and removal of adenomas are important.^[2]^ Identifying risk factors for adenoma may also be useful for preventing CRC.

Periodontitis is common oral disease that affects 10% to 15% of the adult population worldwide.^[3]^ The disease begins as acute inflammation of the gingival tissue and progress toward the gradual destruction of periodontal tissues, including periodontal ligament, cementum, and alveolar bone supporting the teeth.^[4,5]^ The most important and prevalent anaerobic Gram-negative bacteria in the subgingival area involved in the development of periodontitis are *Actinobacillus actinomycetemcomitans*, *Porphyromonas gingivalis*, *Prevotella intermedia*, and *Tannerella forsythensis*.^[6–8]^ These bacteria play an important role in the onset and subsequent development of periodontitis by participating in the formation of periodontal pockets, connective tissue destruction, and alveolar bone resorption through an immnunopathogenic mechanism.^[6]^ Interestingly, in dental plaque of healthy people, many of these bacteria coexist at low levels with high levels of Gram-positive bacteria in the biofilm that develops on the tooth surface.^[9,10]^ Periodontal disease progression is signaled by a shift in the bacterial makeup of the dental biofilm from largely aerobic Gram-positive bacteria to a pathogenic infectious state dominated by anaerobic Gram-negative organisms.^[9]^

Several recent studies have reported that the people with periodontal disease or tooth loss are at higher risk of oral, head, and neck cancer as well as various systemic diseases.^[11–14]^ People with periodontal disease also have a significantly increased risk of gastrointestinal cancer including cancers of the upper aerodigestive tract, esophagus and stomach.^[15,16]^ There is growing awareness of the link between periodontal and systemic inflammatory conditions such as rheumatoid arthritis, atherosclerosis, inflammatory bowel disease (IBD).^[17]^ It is also well known that the smoking, high body mass index (BMI), low physical activity, obesity, and dietary factors are associated with adenoma.^[18–21]^ To our knowledge, no studies have evaluated whether the oral health status is related to the risk of colorectal adenoma, an important risk factor for CRC.^[22,23]^ In this study, we sought to determine whether oral health status, including periodontitis and tooth loss, is related to the risk of colorectal adenoma in a patients who underwent a screening colonoscopy.

## 2 Methods

### 2.1 Study population

We used data from consecutive patients who underwent health screening between January 2013 and October 2015 at the Kangbuk Samsung Hospital, Seoul. The institutional review board at Kangbuk Samsung Hospital approved the research procedure (2017-02-035). All enrolled patients provided informed consent. The enrolled patients underwent various examinations including blood tests, esophagogastroduodenoscopy and colonoscopy. The patients were also asked to complete a standardized scripted health questionnaire that asked about demographic and clinical information including smoking behavior, daily alcohol intake and daily physical activity. The exclusion criteria were: lack of a colonoscopic examination as part of the health screening; a history of colonoscopic examinations. We excluded patients with the history of colonoscopy. Those patients might have had their adenoma removed previously so the previous history of colonoscopy can create a bias in selection; colon and rectal resection; limited results of the examination because of poor bowel preparation or failure of cecal insertion; and missing data.

### 2.2 Definitions

Abdominal obesity was defined as a waist circumference ≥90 cm in males and ≥85 cm in females.^[24]^ We calculated the daily amount of alcohol intake from the information provided by patients in the questionnaire. Alcohol consumption was classified into four levels: none, light (≤1 drink/d or <12.5 g/d), moderate (12.5–30 g/d), or heavy (>30 g/d). We ascertained smoking history with the following questions. First, we asked whether the patient is currently smoking, currently quitting, or has never smoked. Second, if the patient answered positively to the question about current smoking, we asked the patient to state the number of packs smoked a day and categorized their annual cigarette consumption into 5 pack-year categories: 0-9, 10-20, 20-30, 30-40, ≥40 and above. BMI was classified into three categories based on the World Health Organization Asia-Pacific guidelines on body mass index: “normal” (BMI <23 kg/m^2^, “overweight” (23 ≤ BMI < 25 kg/m^2^), “obese” (BMI ≥ 25 kg/m^2^).^[25]^

### 2.3 Colonoscopic examination

All colonoscopic examinations were performed by gastroenterology specialist in internal medicine or general surgery using a video colonoscope (CV-290 or CV260; Olympus Medical Systems Co, Ltd, Tokyo, Japan). The mechanical bowel preparation was accomplished using 4 L polyethylene glycol (Colyte; Taejun Pharm Co Ltd, Seoul, Korea). The withdrawal time of all patients who underwent colonoscopic examination was more than 6 minutes on average. A successful colonoscopic examination required that the colonoscope reached the cecum, which was confirmed when both the appendiceal orifice and the ileocecal valve were identified. The Boston bowel preparation scale was used to assess bowel preparation quality of the right colon, transverse colon, and rectosigmoid colon. The grade of each segment was measured by the endoscopist based on a scale 0 to 3 (0 being poor to 3 being excellent) defined as follows: 0 = unprepared colon segment with mucosa not seen because of solid stool that cannot be cleared; 1 = portion of the mucosa of the colon segment seen, but other areas of the colon segment not seen well because of staining, residual of stool, and/or opaque liquid; 2 = minor amount of residual staining, small fragments of stool, and/or opaque liquid, but the mucosa of the colon segment can be seen well; and 3 = entire mucosa of the colon segment is seen well with no residual staining, small fragments of stool, or opaque liquid.^[26]^

All detected colorectal polyps were biopsied or polypectomized for histopathologic examination, except for multiple polyps in the rectosigmoid area that appeared to be hyperplastic polyps based on endoscopic features consistent with a hyperplastic histology such as small size, sessile shape, pale color.^[27]^ The location and size of polyps were recorded. All polyps were reviewed by gastrointestinal pathologists in accordance with the World Health Organization criteria.^[28]^

### 2.4 Oral examinations

We use both a self-reported oral health questionnaire and oral examination for all patients. There is no standardized definition or clinical criteria for periodontal disease used consistently in the periodontal epidemiological literature, which makes it difficult to compare studies of the association between periodontal disease and other systemic diseases.^[5]^ The centers for Disease Control and Prevention in collaboration with the American Academy of Periodontology has formulated self-report questionnaires that appear to be promising in predicting the prevalence and severity of periodontitis among the adult population.^[29]^ Recent studies reported that sensitivity of periodontitis by self-reported questionnaire was 68% to 80% in nonhealth professionals.^[30,31]^ To improve the quality of questionnaire such as specificity and sensitivity, we used more sophisticated questionnaire and also used oral examinations and radiographs by dentists. The survey questionnaire comprised 25 items about oral health status and habit including past medical history, brushing habit, sugar intake, oral hygiene, fluoride use (Table 1). Five additional items were added to patients who have denture (Table 2). All oral examinations were performed by 2 dentists with over 10 years of extensive experience.

**Table 1:**
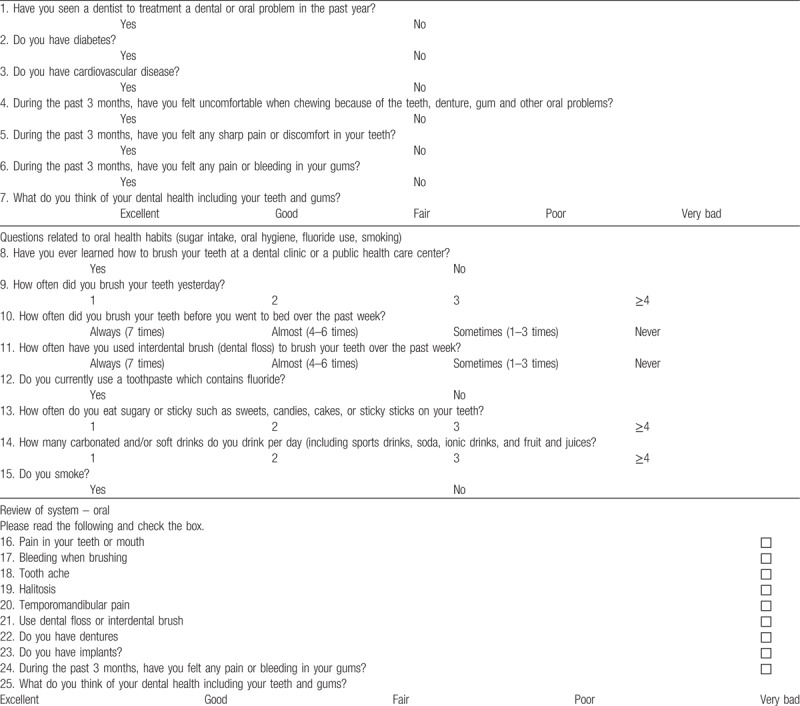
Please read and answer the following questions.

**Table 2:**
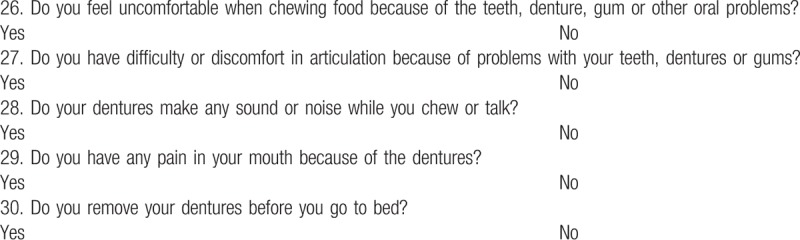
Questions on status of oral functioning (if you use dentures, please answer what you feel while wearing the dentures).

### 2.5 Data analysis

The primary outcome was the relationship between oral health status and the prevalence of colorectal adenoma. Continuous variables were analyzed using the Student *t* test, and categorical variables were compared using the Chi-squared test. All data are presented as the mean ± standard deviation or numbers with percentage. Multiple logistic regression analyses were used to control the confounding variables and to assess the independent risk factors for colorectal adenoma. The odd ratio (OR) and the 95% confidence intervals (CI) were used in the logistic regression analysis. *P*-value ≤.05 was considered to be statistically significant. The data were analyzed using Stata (version 13; StataCorp LLC, College Station, TX).

## 3 Results

During the study period, 239,455 patients underwent health screening that included colonoscopy and multiple examinations, and completed the health questionnaire. A total 93,692 patients were excluded for reasons: 37,702 had a previous colonoscopy; 7623 had incomplete data; 5399 had an inadequate bowel preparation; and 97 had a previous history of colorectal surgery. A total 42,871 patients met the eligibility criteria for inclusion in this study. Table 3 shows the baseline characteristics of patients. Their mean age was 39.3 ± 8.7, 70.4% were male, 6.5% had hypertension, and 1.9% had diabetes mellitus. The median BMI was 23.5 ± 3.3 kg/m^2^. The prevalence of adenoma was 12%. Patients with an adenoma had larger abdominal circumference and high blood pressure, low-density protein concentration, and HbA1c level. A higher percentage of patients with adenoma had diabetes and hypertension.

**Table 3:**
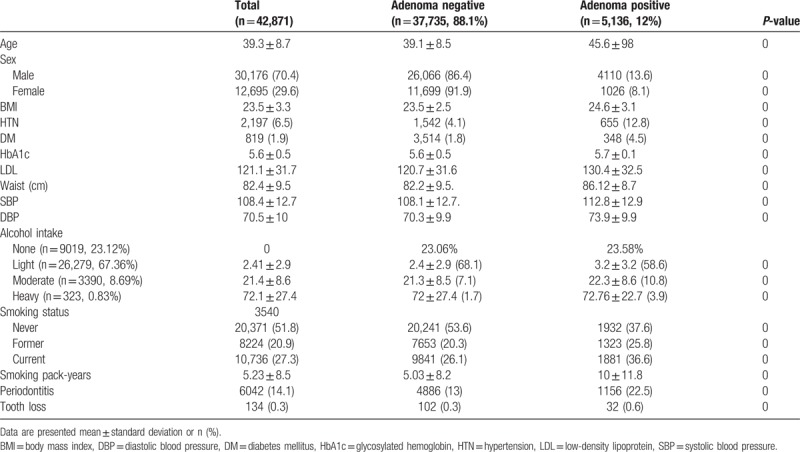
Baseline characteristics and association of potential factors with adenomas.

Table 4 shows the results of both the univariate and multivariable logistic regression analyses for identifying risk factors associated with colorectal adenoma. In the multivariate analysis, colorectal adenoma was associated with age >45 years, obesity (BMI > 25 kg/m^2^), current smoking, >10 smoking pack-years, and moderate-heavy daily alcohol intake. In the logistic regression analysis, after controlling for age, BMI, smoking pack-years, and daily alcohol intake, periodontitis was associated with a significantly increased risk of adenoma (OR = 1.29, 95% CI 1.18–1.4; *P* < .001). Tooth loss was an individual risk factor that was significantly associated with adenoma in the logistic regression analysis (OR = 1.67, 95% CI = 1.04–2.68; *P* < .001).

**Table 4:**
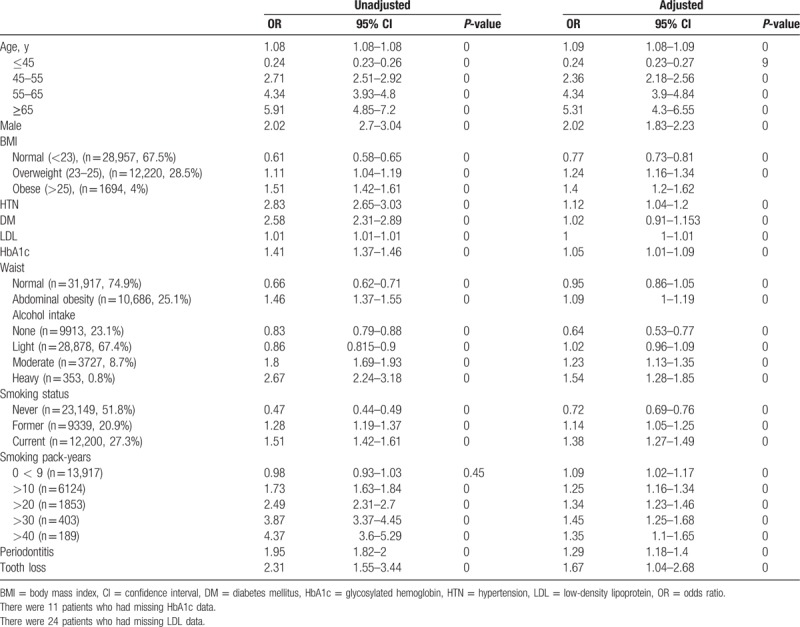
Association of potential risk factors and colorectal adenoma.

## 4 Discussion

In this large cross-sectional study, we examined the relationship between oral health status and risk of colorectal on colorectal adenoma. Over the past few years, a growing number of studies have reported on a relationship between oral health, including periodontitis and tooth loss, and many systemic diseases.^[3,13,14,32]^ Periodontal infection contributes to diabetes by disrupting glycemic control.^[33]^ Respiratory disease such as chronic obstructive pulmonary disease and mother of a preterm child are also known to be related to severe periodontitis.^[34,35]^ One possible explanation for this relationship is that the oral cavity can be a reservoir for systemic dissemination of pathogenic bacteria and their toxins, which may lead to infections and inflammation in distant body sites.^[36]^

Many studies have examined the associations between periodontal disease and cancers. Periodontal disease and tooth loss are risk factors for several solid tumors including those of the breast, lung, pancreas, prostate, and kidney, although the relationship between periodontal disease and risk for hematological cancers has not been observed consistently.^[11,13]^ In general, the most studies suggest that tooth loss is related to the risk of CRC, although the results of studies of the relationship between periodontal disease and CRC are mixed. Some studies of the associations between various forms and history of periodontal disease have found no association between periodontal disease and the risk of CRC.^[13,37]^ By contrast, 1 study reported that women with moderate or severe periodontal disease were at a higher risk of CRC compared with those without history of periodontitis.^[12]^ As noted earlier, previous studies have investigated mainly CRC, which is a late stage of adenoma-carcinoma sequence of colon and rectal cancer. The objective of our study was to examine the relationship between oral health and the risk of colorectal adenoma, which begins with the transformation of normal mucosal cells. Surprisingly, we found a strong association between oral health and colorectal adenoma. In this population, periodontitis and tooth loss were associated with colorectal adenoma to the same extent as were a moderate alcohol intake or smoking >30 pack-years.

Periodontal disease generally refers to inflammatory pathologic state of the gingiva and the supporting structures of the periodontium, which include the gingiva, alveolar bone, periodontal ligament, and cementum.^[4]^ In addition to the 4 important bacteria, mentioned earlier, other Gram-negative bacteria including *Prevotella* and *Fusobacterium* spp also play an important role in this disease process. Singly, these microbial species do not cause the destructive events involved in the progression of periodontal disease, and the etiology requires the concerted interaction of these microorganisms to establish their niches in the oral cavity.^[38,39]^

Some of these bacteria have been reported to be associated with development of IBD. *Fusobacterium nucleatum* has a well-known effect on the gastrointestinal system and has been isolated from tissues involved in numerous inflammatory processes, including sinusitis, endocarditis, septic arthritis, tonsillitis and abscesses of the brain, skin and liver.^[40,41]^ Human trials and animal experiments have confirmed the presence of *P gingivalis* in liver tissues.^[42]^ This microorganism can adhere to and invade different types of host cells, including human epithelial and endothelial cells, by expressing the surface adhesin FadA.^[36]^ FadA binding to vascular endothelial cadherin causes the latter to translocate from cell–cell junctions to intracellular compartments, which increases endothelial cell permeability and allows bacteria to penetrate.^[43]^*F nucleatum*is invasive by itself and has also been shown to facilitate both intra- and intercellular invasion by other species, such as *Streptococcus cristatus* and *Escherichia coli*.^[44]^

Recent studies have shown similarities in the pathogenesis of periodontitis and IBD. Both periodontitis and IBD involve hypersensitivity to commensal bacteria, and antibodies against various bacterial plaque components are present in the serum of IBD patients with periodontitis.^[45]^ Oral bacteria such as *F nucleatum* and *Campylobacter concisus* seem to be important to the development of IBD.^[46,47]^ Koutsochristou et al studied 55 youths, aged 4 to 18 years, on remission from a single outpatient IBD clinic and 55 matched systemically healthy controls who were assessed prospectively in a dental practice.^[48]^ The youths with IBD had a significantly (*P* < .001) higher chance of having decayed, missing, or filled teeth and of needing periodontal treatment. The results of that case–control study suggest that, despite similar oral hygiene status as healthy youths, children and adolescents with IBD have a higher frequency of dental caries, more clinical signs of gingival inflammation, and greater need for periodontal treatment.^[48]^

Patients with long-standing ulcerative colitis and Crohn disease have an increased risk of developing CRC.^[49]^ IBD is an important risk factor for CRC because it disturbs the host's mucosal immunity of host. Unlike the well-known adenoma-carcinoma sequence, the mechanism of IBD-related colon cancer involves the inflammation-dysplasia-carcinoma sequence.^[50]^ The cancer caused by IBD-related colitis is called colitis-associated cancer (CAC). The processes of aberrant crypt foci, polyps, adenomas, and carcinomas are the same for CRC and CAC, but CAC causes well-defined inflammation, damage, and dysplasia without affecting the glands.^[51]^ Patients with IBD are at an increased risk of developing colorectal advanced neoplasia, including colorectal high-grade dysplasia and CRC.^[52,53]^ Considering the findings of previous studies, we speculate that oral health is closely related to the development of IBD and that this systemic inflammatory reaction may could subsequently affect the development of CRC by initiating dysplasia-carcinoma sequence in the colon and rectum.

Our study has adequate statistical power because of large sample size. However, our study is not free of some limitations. First, we could not identify severity of periodontitis. The classification of periodontal disease requires careful evaluation of the extent and severity (none, mild, moderate, severe) by radiographs, dental probe, or Vernier calipers.^[54,55]^ It was not possible for us to perform dental check-ups using calipers and micrometers for every patient due to the time-consuming and costly nature of large-scale health screening. Thus, we tailor-made periodontal disease questionnaire to suit our study's sample characteristics. We think that our research has improved specificity because we used more sophisticated questionnaire as well as oral examinations. Second, we did not collect data about the specific characteristics of the adenomas (ie, size, location, and number). This makes it difficult to determine the stage of the adenomas (early or advanced) in the adenoma-carcinoma sequence. Third, our study used a cross-sectional design and included only a single ethnic group. There is no standardized and valid population-based case definition of periodontitis, and it is difficult to compare the epidemiological data for periodontal disease because of the inconsistencies in the definitions and methodology used. A previous study reported that the prevalence of periodontitis is high. That is, about 10% of the global population is affected by severe periodontitis.^[56]^ In the United States, a recent study confirmed the high prevalence of periodontitis in US adults aged over 30 years and found that almost 50% are affected.^[57]^ A cross-sectional analysis performed in Europe, in France, the United Kingdom, and Denmark, reported that the prevalence of periodontitis ranges from 15% to 82%.^[58–60]^ Because of differences in the populations and social environment between studies, it may not be possible to generalize the interpretations of our result. Therefore, we plan to investigate the association between oral health and CRC risk in patients using the Community Periodontal Index developed by the WHO for use in epidemiological screening. Fourth, we were unable to analyze family history from patients. Regarding the National Colon Cancer Screening, only the fecal occult blood test is free of charge, and the cost of colonoscopy is fully borne by patients in South Korea. Thus, it is difficult to analyze the family tree due to insufficient data. Fifth, as found out by many studies, socio-economic status has an influence on severity of CRC and adenoma in the United States.^[61]^ However, we were not able to include socio-economic status of our sample individuals in our analysis due to the lack of data availability. Finally, another limitation of this study is that almost 8% of the patients have missing information on smoking and alcohol intake data. As a result, these observations are dropped in the multivariate analysis. Hence, these missing observations could potentially impact the significance of the association we study.

## 5 Conclusion

Although periodontitis is a benign disease, the results of this study suggest that the incidence of colorectal adenoma in patients with periodontitis and tooth loss is high. When compared with other risk factors, the OR of periodontitis for the risk of adenoma was similar to that of smoking 10 to 20 pack-years or a moderate amount of alcohol intake. Considering that oral health is closely related to systemic inflammatory conditions such as IBD, physicians should be aware of a possible relationship between oral health and gastrointestinal mucosal status, including crypt foci, polyps, adenoma, and CRC, and that poor oral health may be a risk factor for colorectal adenoma. We suggest that more frequent use of oral examinations with radiography may be helpful for population-based health screening. Further studies of the association between oral health and gastrointestinal neoplasia may help to identify and prevent gastrointestinal malignancy.

## Author contributions

**Conceptualization:** Donghyoun Lee, Hyung Ook Kim.

**Data curation:** Donghyoun Lee, Kyung Uk Jung.

**Formal analysis:** Donghyoun Lee.

**Funding acquisition:** Donghyoun Lee, Kyung Uk Jung.

**Investigation:** Donghyoun Lee, Kyung Uk Jung.

**Methodology:** Donghyoun Lee, Kyung Uk Jung.

**Project administration:** Donghyoun Lee.

**Resources:** Donghyoun Lee.

**Software:** Donghyoun Lee.

**Supervision:** Donghyoun Lee.

**Validation:** Donghyoun Lee, Hunddai Kim.

**Visualization:** Donghyoun Lee, Ho-kyung Chun.

**Writing – original draft:** Donghyoun Lee.

**Writing – review & editing:** Donghyoun Lee, Hyung Ook Kim.
